# Research and Optimization of a Digital Model of a Tracked Vehicle Hydraulic Braking System

**DOI:** 10.3390/ma19081620

**Published:** 2026-04-17

**Authors:** Zhiqiang Liu, Kun Yang, Cenbo Xiong, Zhiqiang Zeng, Liang Yu, Yu Zhou, Songquan Li

**Affiliations:** 1 School of Intelligent Manufacturing Engineering, Shanxi College of Technology, Shuozhou 036000, China; 2 School of Energy and Power Engineering, North University of China, Taiyuan 030051, China; 3 School of Mechanical Engineering, Beijing Institute of Technology, Beijing 100081, China

**Keywords:** tracked vehicle, hydraulic braking system, digital model, braking performance

## Abstract

**Highlights:**

- A high-precision digital model of a tracked vehicle hydraulic braking system is established.
- Simulation accuracy exceeded 95% under different braking levels.
- Optimizing component design parameters can improve braking performance.

**Abstract:**

Due to the complex operating environment of tracked vehicles, experimental braking tests using real vehicles are typically costly and time-consuming. Furthermore, limitations in testing environments make it difficult to comprehensively evaluate a system’s braking performance across diverse operating scenarios. To overcome these limitations, this paper proposes the construction of a high-precision digital model to simulate the real braking process of tracked vehicles in a virtual environment and validates the model through experiments. The results show that braking pressure changes continuously and proportionally with the pedal angle, the system response time is less than 0.3 s, braking pressure builds up rapidly, and the output process is smooth, with no significant overshoot. Under different braking percentage conditions, the simulation accuracy of both braking pressure and response time exceeds 95%, indicating that the established model accurately reflects actual braking performance and provides a theoretical basis for optimizing tracked vehicle braking systems. Finally, by rationally designing the parameters of the accumulator and electro-hydraulic proportional valve and reducing the brake cylinder volume, it is possible to improve braking performance. This provides a theoretical basis for the optimization of tracked vehicle braking systems.

## 1. Introduction

Under complex terrain and heavy-load operating conditions, tracked vehicles have been widely used in a variety of fields, including industrial and agricultural equipment, mining machinery, military equipment, and special transport platforms, owing to their strong traction capability, superior adhesion, low ground contact pressure, and excellent off-road mobility [1,2,3,4,5]. However, compared with wheeled vehicles, tracked vehicles are generally characterized by greater vehicle mass, harsher operating environments, and more severe load variations, which subject their braking systems to more demanding operating conditions [6]. Particularly in the context of continuing advances toward higher speeds, greater mobility, and heavier load capacity, the dynamic response capability and reliability of braking systems have become key factors affecting vehicle safety and operational performance. If a rational structural layout and proper parameter matching are not achieved during the braking system design process, problems such as insufficient braking capacity, excessive temperature rise in the hydraulic system, and accelerated wear of friction pairs may occur, ultimately impairing braking smoothness and safety, and potentially leading to brake failure [7,8,9].

In view of the complex operating environment, significant load fluctuations, and the high-frequency and strongly stochastic nature of the braking process in tracked vehicles, it is difficult to achieve a comprehensive understanding of braking system performance solely through empirical design and limited experimental approaches [10]. Therefore, it is necessary to establish a high-fidelity digital model of the braking system to simulate the actual braking process in a virtual environment, thereby enabling the prediction and evaluation of braking performance and guiding the optimization of key design parameters.

In recent years, researchers have conducted extensive investigations into the optimization of structural parameters of hydraulic valves and the design of braking systems, and a certain degree of progress has been made. Existing studies indicate that hydraulic pipeline parameters exert a significant influence on braking system performance. Stosiak et al. [11], through theoretical and experimental analyses, found that a specific resonant length exists in hydraulic pipelines, at which outlet pressure pulsations are significantly amplified, thereby inducing mechanical vibrations in the pipeline. Albatlan et al. [12] showed that mismatched inner diameters of brake lines on the same axle lead to uneven brake force distribution, causing asynchronous wheel lockup and reduced braking efficiency; thus, consistent pipe diameters should be ensured in the design. Yang et al. [13] established a pressure response model considering heat transfer and friction in pipelines, which shows good agreement with experimental data; however, its accuracy is limited by the neglect of local resistance losses (e.g., pipe bends). Building on this, Meng et al. [14] developed a nonlinear model incorporating pipeline characteristics and valve–pipeline bidirectional coupling, which was validated by full-system experiments. Regarding the design and matching of hydraulic braking system parameters, Yu et al. [15] analyzed the effects of key parameters on hydraulic braking dynamics via Simulink–AMESim co-simulation, showing that increasing the valve port area and fluid bulk modulus improves system response performance. Gao et al. [16] and Vaughan et al. [17] established accurate nonlinear dynamic models of hydraulic valves and verified their accuracy through experiments, thereby providing a theoretical basis for studying the input–output characteristics of hydraulic valves. Yang et al. [18] reported that increasing the spool diameter of the brake valve leads to a closing delay without affecting the opening time, whereas variations in the spool diameter of the rear axle brake valve may result in a delayed opening response. Chen et al. [19] established a full hydraulic braking system model based on AMESim and concluded that increasing the spool diameter of the pressure control valve within the charging valve reduces the upper limit of the charging pressure, whereas increasing the valve seat angle raises the lower limit of the charging pressure, thereby providing a theoretical basis for the design and parameter matching of hydraulic valves. Tan et al. [20] were the first to consider the effects of multi-factor coupling conditions on the analysis and optimization of hydraulic braking systems from a systemic perspective. Their work achieved simultaneous improvements in both system energy consumption and the number of braking operations. Following the implementation of the optimization process, a 32.8% reduction in energy consumption was observed, while the number of braking operations increased by 10%. Tan et al. [21] and Li et al. [22] adopted an orthogonal experimental design method to investigate multiple factors affecting braking system performance, thereby obtaining an optimal parameter combination that significantly improved vehicle braking performance. Kosek et al. [23] employed a CFD-based approach to demonstrate that a proportional directional valve spool with six triangular notches and a 10° control-edge chamfer achieves a linear relationship between spool opening and flow rate, thereby providing a useful reference for proportional valve design. However, the study did not consider the flow characteristics during dynamic spool motion, which limits its applicability. Chen et al. [24] introduced upper and lower circular arcs at the spool shoulder and concave corners of an electro-hydraulic proportional valve and adopted inclined inlet and outlet flow passages. These modifications suppress vortex formation and reduce turbulent kinetic energy, thereby decreasing energy loss and providing an effective approach for low-energy proportional valve design. To address performance and reliability issues caused by excessive and imbalanced hydraulic force in valves, Yang et al. [25] and Liu et al. [26] reduced steady-state hydraulic force and power consumption by optimizing the valve body structure. Wang et al. [27] and Li et al. [28] proposed an asymmetric combined flow-guiding spool structure that significantly reduces axial steady-state hydraulic force while suppressing the growth of radial unbalanced forces by controlling inlet and outlet jet characteristics, thereby improving spool motion stability and system reliability. However, these studies only focus on steady-state hydraulic forces, and further investigation of transient responses under complex operating conditions is still required. Regarding system control strategies, Yang et al. [29] developed a mechanical–hydraulic co-simulation model and applied fuzzy PID control to mitigate braking impact and improve response, thereby reducing brake disc axial oscillations. However, the study focuses primarily on simulation and lacks experimental validation of the model. To address the issues of response lag and insufficient control accuracy associated with PID control in traditional hydraulic braking systems, Wu et al. [30] and Zhou et al. [31] proposed different improved control strategies, both of which significantly enhanced system response speed, control accuracy, and disturbance rejection capability, thereby improving vehicle braking performance.

Existing research primarily focuses on the parameter design and matching of individual hydraulic components and on system control strategies, with relatively limited attention being paid to high-precision modelling of hydraulic braking systems. Although Lundin et al. [32] and Viselga et al. [33] developed braking analysis models capable of evaluating vehicle braking performance to some extent, these models do not fully account for the complex nonlinearities of the system and are therefore mainly suitable for preliminary validation during the early design stages, rendering them insufficient for accurate performance prediction and structural optimization. Against the backdrop of the aforementioned research, this paper takes a certain model of tracked vehicle as its subject of study. It constructs a high-precision digital model that accounts for the actual structural parameters of various hydraulic components and complex nonlinear factors to simulate real-world braking conditions. This enables the prediction and evaluation of the braking system’s performance. The model’s accuracy is then validated through experiments, providing a reference for the design optimization and performance analysis of tracked vehicle braking systems.

## 2. Composition and Working Principle of the Hydraulic Brake System

**Figure 1:**
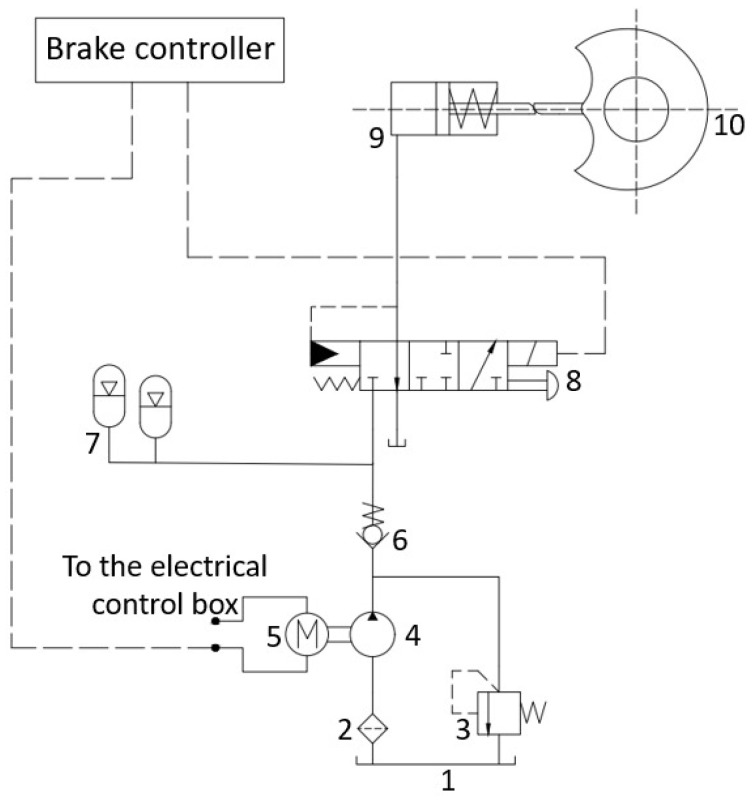
Schematic diagram of the hydraulic brake system. 1—Oil tank. 2—Filter. 3—Relief valve. 4—Fixed-displacement pump. 5—Motor. 6—Check valve. 7—Accumulator. 8—Electro-hydraulic proportional valve. 9—Brake cylinder. 10—Brake.

Figure 1 illustrates the working schematic of a hydraulic braking system for a certain type of tracked vehicle.

During the operation of a tracked vehicle, when the driver depresses the brake pedal, a corresponding angular displacement is generated, which is detected in real time by a pedal angle sensor and converted into an electrical signal transmitted to the braking controller. Based on this electrical signal, the braking controller outputs a corresponding control current that drives the solenoid to generate an electromagnetic force acting on the spool of the electro-hydraulic proportional valve, causing it to move within the valve body and thereby vary the valve port opening. As the spool position changes, the pressures in the braking hydraulic circuits on both sides are adjusted, enabling the braking pressure to vary continuously and proportionally with the pedal angle. The braking pressure is transmitted via the brake piston push rod to the brake mechanism, driving the piston to compress the friction linings and generate braking torque proportional to the applied pressure, thereby achieving braking of the tracked vehicle.

## 3. Mathematical Models of Various Hydraulic Components

The digital model of the braking system is based on a mathematical model and, while adhering to the system’s operating mechanism, is capable of performing dynamic simulations under complex braking conditions. Based on a systematic analysis of the structural composition and working principle of the tracked vehicle braking system, a mathematical model is first established to characterize the dynamic relationships among hydraulic components as well as their pressure–flow characteristics.

To ensure the efficiency of model calculation and meet the needs of system dynamic characteristic analysis, the following assumptions are made in this paper:

(1) The effect of temperature change on hydraulic oil viscosity is ignored.
(2) Oil leakage caused by the pipeline connection is not considered.
(3) The flow of hydraulic oil in the accumulator is regarded as laminar flow.
(4) The filling process of the accumulator is slow, and the changes in gas pressure and volume can be approximated as an isothermal process; the discharge of hydraulic oil from the accumulator is fast, and the discharge process can be regarded as an adiabatic process.
(5) The mathematical model of the electro-hydraulic proportional valve and brake cylinder is simplified, and structural nonlinear factors such as dead zone and hysteresis are ignored.

### 3.1. Hydraulic Pump

Equation (1) represents the output flow rate $Q_{pump}$ of the hydraulic pump:

(1) $$Q_{pump}=\eta \times V_{pump}\times n$$

In the equation, η is the volumetric efficiency of the hydraulic pump; $V_{pump}$ is the displacement of the hydraulic pump; and n is the motor speed.

### 3.2. Accumulator

Equation (2) represents the gas volume of the accumulator:

(2) $$V_{gas}=V_{acc0}\;\times {\left(\frac{P_{acc0}}{P_{\mathrm{gas}}}\right)}^{\left(\frac{1}{\gamma }\right)}$$

In the equation, $P_{acc0}$ and $V_{acc0}$ are the initial absolute gas pressure and volume in the accumulator, respectively; $P_{\mathrm{gas}}$ and $V_{gas}$ represent the absolute pressure and volume of the gas within the accumulator, respectively; and γ is the polytropic exponent of the gas. $P_{gas}$ is defined as the sum of the accumulator oil port pressure $P_{accout}$ and the atmospheric pressure $P_{atm}$.

(3) $$P_{gas}=P_{accout}+P_{atm}$$

Differentiating Equation (2) with respect to time, we get

(4) $$\frac{dV_{gas}}{dt}=-\frac{V_{gas}}{\gamma P_{gas}}\frac{dP_{gas}}{dt}$$

The relationship between the accumulator oil port flow rate $Q_{accout}$ and the change in gas volume is given as follows:

(5) $$Q_{accout}=-\frac{dV_{\mathrm{gas}}}{dt}$$

Since the compressibility of the hydraulic fluid is negligible compared with that of the gas, the time derivative of the oil pressure is equal in magnitude to that of the gas pressure.

(6) $$\frac{dP_{accout}}{dt}=\frac{dP_{gas}}{dt}$$

Equation (6) represents the relationship between the accumulator flow rate and the oil port pressure.

(7) $$Q_{accout}=\frac{dP_{accout}}{dt}\;\frac{V_{gas}}{\gamma (P_{accout}+P_{atm})}$$

### 3.3. Electro-Hydraulic Proportional Valve

**Figure 2:**
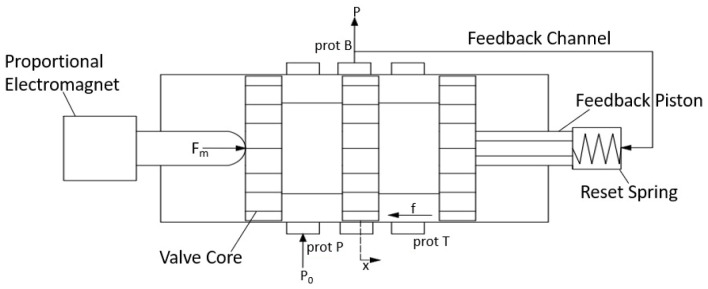
Structural diagram of an electro-hydraulic proportional valve.

Figure 2 presents a schematic structural diagram of the electro-hydraulic proportional valve.

When the tracked vehicle is not braking, load port B of the electro-hydraulic proportional valve is connected to the return port T. At the onset of braking, the braking controller supplies a current I to the proportional solenoid of the electro-hydraulic proportional valve, generating an electromagnetic force $F_{m}$ that drives the spool to move gradually rightward against the spring force, allowing for pressurized oil from the accumulator to flow from port P through port B to the brake cylinder, while the outlet pressure p acts on the right end of the spool via the feedback chamber. As the outlet pressure p increases progressively, when the combined force exerted by the feedback chamber pressure and the spring force exceeds the electromagnetic force, the spool is driven leftward until a new equilibrium position is reached, at which point the hydraulic connection between ports P and B is cut off and the braking pressure remains constant.

Equation (8) represents the force balance equation of the valve spool when the electro-hydraulic proportional valve port is open.

(8) $$F_{m}-pA=m\ddot{x}+(c_{v}+c_{s})\dot{x}+(K_{v}+K_{s})(x_{0}+x)+f$$

In the equation, $F_{m}$ is the electromagnetic force; p is the outlet pressure; A is the effective piston area of the feedback chamber; m is the spool mass; x is the spool displacement; $c_{v}$ is the viscous damping coefficient of the spool; $c_{s}$ is the hydrodynamic damping coefficient; $K_{v}$ is the stiffness of the return spring; $K_{s}$ is the hydrodynamic stiffness; $x_{0}$ is the pre-compression of the return spring; and f is the friction force acting on the spool.

Equation (9) represents the valve port flow rate.

(9) $$Q=C_{d}A(x)\sqrt{\frac{2\left|p_{0}-p\right|}{\rho }}$$

In the equation, $C_{d}$ is the discharge coefficient of the valve port; A(x) is the effective flow area; $p_{0}$ is the supply pressure of the hydraulic system; and ρ is the density of the hydraulic oil.

### 3.4. Brake Cylinder

During the braking process of a tracked vehicle, the braking pressure generated by the electro-hydraulic proportional valve acts on the piston of the brake cylinder, driving the friction linings and friction disc into contact to generate friction. As the braking pressure increases, the braking torque of increases proportionally, thereby converting the vehicle’s kinetic energy into thermal energy and achieving deceleration.

Equation (10) represents the dynamic equation of the brake cylinder.

(10) $$pA_{2}=m_{2}\frac{d^{2}x_{2}}{dt^{2}}+c_{V2}\frac{dx_{2}}{dt}+k(x_{20}+x_{2})+f_{2}$$

In the equation, $A_{2}$ is the effective piston area of the brake cylinder; $m_{2}$ is the piston mass; $x_{2}$ is the piston displacement; $c_{V2}$ is the equivalent damping coefficient of the brake cylinder piston; k is the spring stiffness; $x_{20}$ is the spring pre-compression; and $f_{2}$ is the friction force acting on the brake cylinder piston.

Equation (11) represents the flow continuity equation of the brake cylinder.

(11) $$Q_{2}=A_{2}\frac{dx_{2}}{dt}+\frac{V_{2}}{E}\frac{dp}{dt}+C_{2}p$$

In the equation, $V_{2}$ is the volume of the working chamber of the brake cylinder piston; E is the bulk modulus of elasticity of the hydraulic fluid; and $C_{2}$ is the leakage coefficient of the brake cylinder.

### 3.5. Pipeline

Equation (12) represents the differential equation for calculating the outlet pressure of the pipeline.

(12) $$\frac{\partial P_{line}}{\partial t}=-\frac{B}{A_{line}}\cdot \frac{\partial Q_{line}}{\partial x}$$

In the equation, $A_{line}$ is the cross-sectional area of the pipeline; B is the equivalent bulk modulus considering the compressibility of both the hydraulic fluid and the pipeline; and $E_{wall}$ is the bulk modulus of elasticity of the pipeline wall material.

(13) $$B={\left(\frac{1}{E}+\frac{1}{E_{wall}}\right)}^{-1}$$

Equation (14) represents the calculation formula for the inlet oil flow rate of the pipeline.

(14) $$Q_{line}=A_{line}\sqrt{\frac{2d_{line}|\Delta P-\rho Lgsin(\theta )|}{L\rho f_{f}}}$$

In the equation, $d_{line}$ is the pipeline diameter; L is the pipeline length; θ is the vertical inclination angle of the pipeline; and $f_{f}$ is the pipeline resistance coefficient.

## 4. Model Establishment and Simulation Calculation

**Figure 3:**
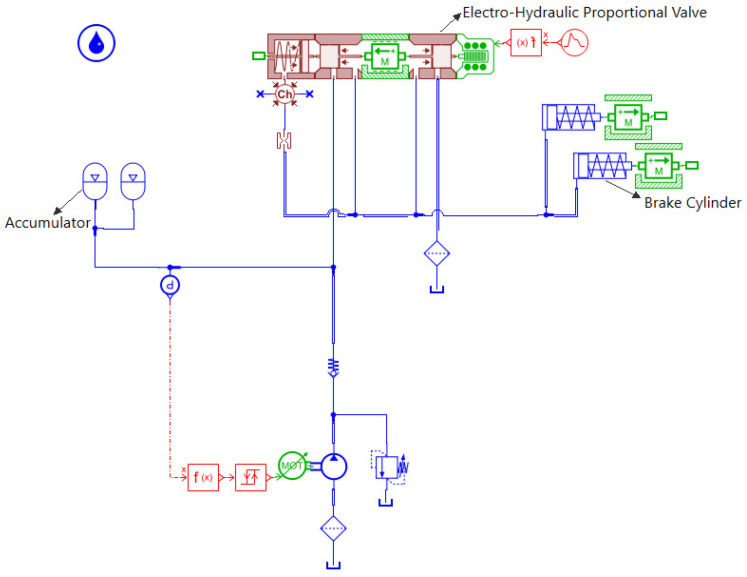
Simulation model of a hydraulic brake system.

Based on the mathematical models of the aforementioned hydraulic components and considering the working principle and actual structural configuration of the tracked vehicle hydraulic braking system, a simulation model was established in AMESim, as shown in Figure 3. The parameters of each hydraulic component are configured according to their actual structural characteristics and design specifications to ensure the accuracy of the model.

### 4.1. Dynamic Operating Conditions

#### 4.1.1. Proportional Signal

**Figure 4:**
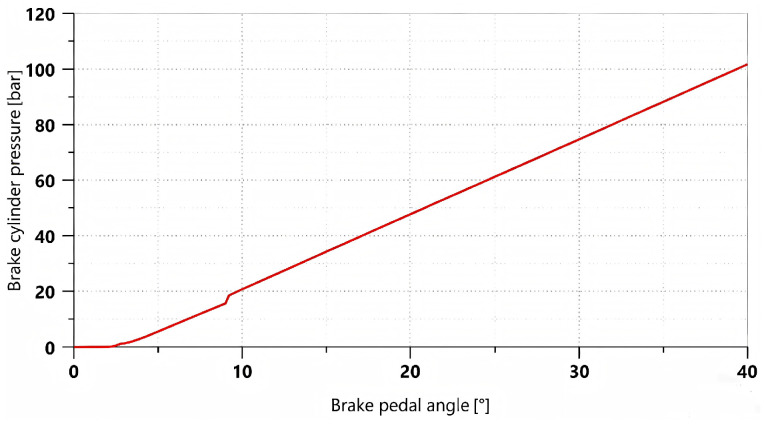
Brake pressure curve varying with pedal opening.

A linearly varying proportional control signal was applied to simulate the gradual depression of the brake pedal, allowing the braking percentage to increase from 0 to 100% within 2 s. The variation curve of brake cylinder pressure with respect to the pedal opening is shown in Figure 4.

As shown in Figure 4, during the initial stage, when the brake pedal rotation angle increases from 0° to approximately 2°, there is essentially no pressure in the brake cylinder due to the pedal’s free travel. As the pedal rotation angle further increases, the braking system begins to build pressure. The brake cylinder pressure increases approximately linearly with the pedal rotation angle and reaches its maximum when the pedal rotation angle reaches 40°, at which point the pressure is 101.72 bar.

#### 4.1.2. Trapezoidal Signal

**Figure 5:**
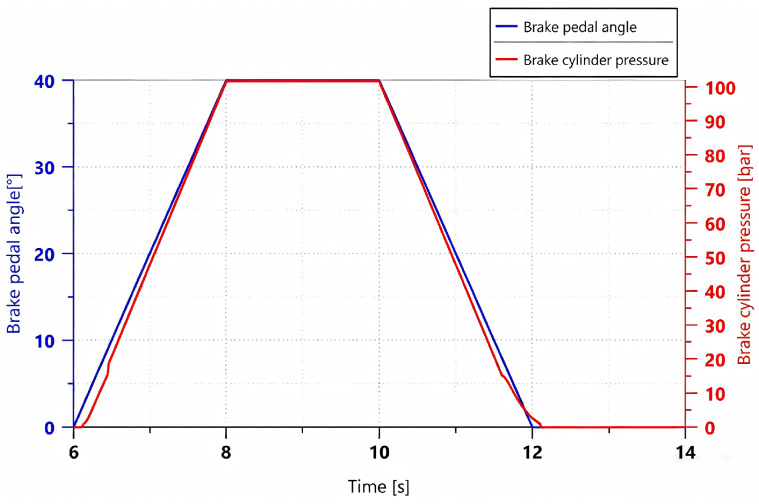
Variation curves of brake pedal rotation angle and brake cylinder pressure under a trapezoidal control signal.

A trapezoidal control signal was applied to simulate the process in which the brake pedal is gradually depressed within 2 s, maintained for 2 s until a steady state is reached, and then gradually released over the subsequent 2 s. Under this operating condition, the relationship between pedal rotation angle and brake cylinder pressure is shown in Figure 5.

As shown in Figure 5, the simulation results indicate that the braking system has good following characteristics for trapezoidal input signals. During the stages of brake pressure buildup, maintenance, and release, the system is capable of generating braking pressure in accordance with the braking demand signal. The overall response process is stable with a relatively fast response, and no significant deviation is observed. In the later stage of pressure release, when brake cylinder pressure decreases to below 7 bar, the pressure decay rate shows a slight lag relative to the change in pedal rotation angle. This is because the pressure difference between the brake cylinder and the oil reservoir gradually decreases during this stage, resulting in a reduced hydraulic oil flow rate and, consequently, a more gradual pressure decline. Nevertheless, the system is still able to discharge all hydraulic oil within 0.2 s, ensuring normal vehicle operation.

### 4.2. Steady-State Operating Conditions

**Figure 6:**
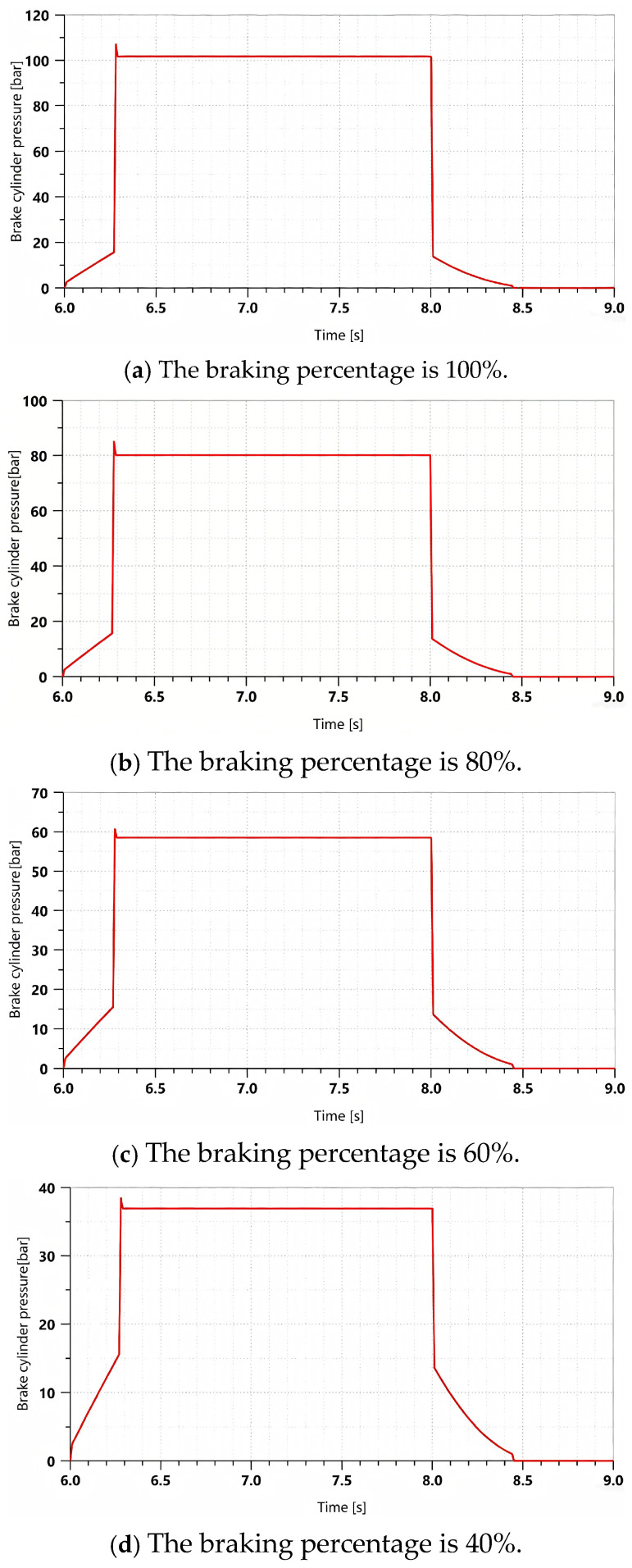
Brake pressure variation curve under a rectangular signal with different brake percentages.

Figure 6 shows the variation curve of braking pressure when a rectangular signal is applied to simulate a single instantaneous full depression of the brake pedal, which is maintained for 1 s and then rapidly released.

In this study, the moment when the braking pressure first reaches its steady-state value is defined as the system response time. As shown in Figure 6, the response time of the braking system is within 0.3 s, indicating that the system responds rapidly to braking commands and can establish effective braking pressure within a short time. Meanwhile, no significant overshoot or pressure fluctuation occurs throughout the braking process, and the pressure remains relatively stable. During the stage of brake release, the hydraulic oil in the brake cylinder can be completely discharged into the oil tank within 0.5 s, enabling rapid brake release and ensuring normal vehicle operation.

**Figure 7:**
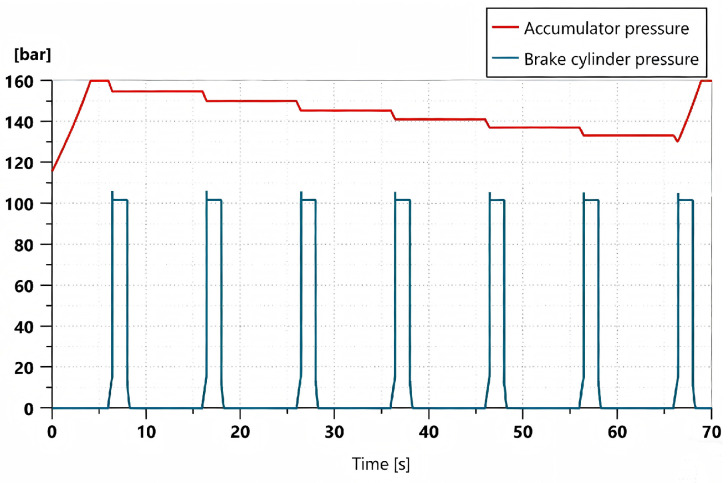
Change curve of the accumulator pressure and brake cylinder pressure.

As the power source of the hydraulic braking system, the accumulator is required to provide sufficient braking pressure; therefore, it is necessary to investigate its pressure variation during the braking process. Figure 7 presents the variation curves of accumulator pressure and brake cylinder pressure when a full brake pedal depression is applied instantaneously, maintained for 2 s, then released instantly, and this process is repeated seven times.

As shown in Figure 7, the accumulator pressure decreases by approximately 4.5 bar during each braking event. After six consecutive braking operations, the accumulator pressure drops below 130 bar, at which point the accumulator begins to recharge. The recharging process takes approximately 3 s, which satisfies the requirement for rapid replenishment of the braking system.

### 4.3. Random Operating Conditions

**Figure 8:**
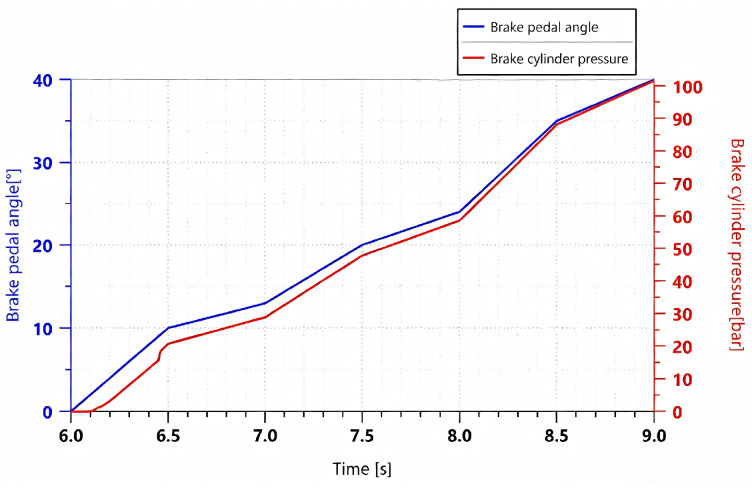
Curve of pedal angle versus brake cylinder pressure under a random signal.

To simulate whether the braking system of a tracked vehicle can generate effective braking pressure under more complex operating conditions, six proportional input signals were randomly set, corresponding to pedal angles of 10°, 13°, 20°, 24°, 35°, and 40°. Each segment increased to the corresponding angle within 0.5 s. Figure 8 shows the pedal angle and braking pressure variation curves.

As shown in Figure 8, with changes in the pedal angle, the braking pressure responds promptly, exhibiting good dynamic following characteristics. The pressure build-up and variation processes are smooth, the response is fast, and no significant fluctuations occur, indicating high overall stability.

## 5. Experimental Verification

**Figure 9:**
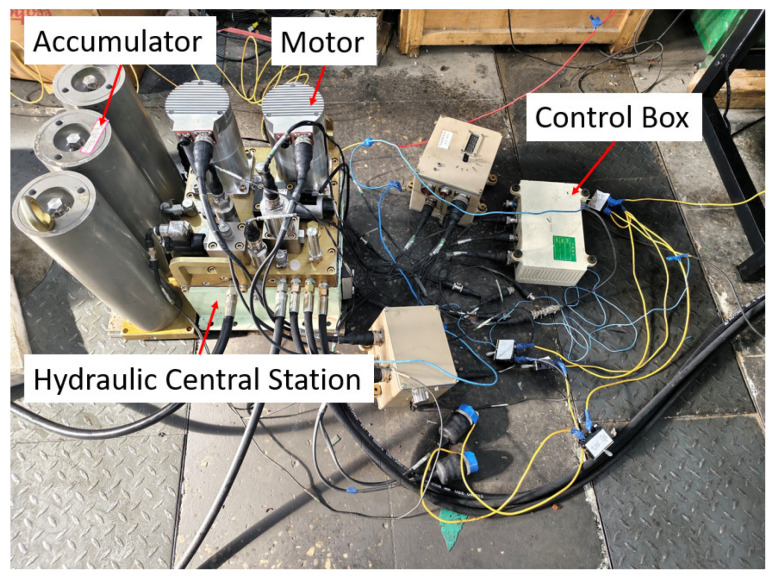
Test bench.

**Figure 10:**
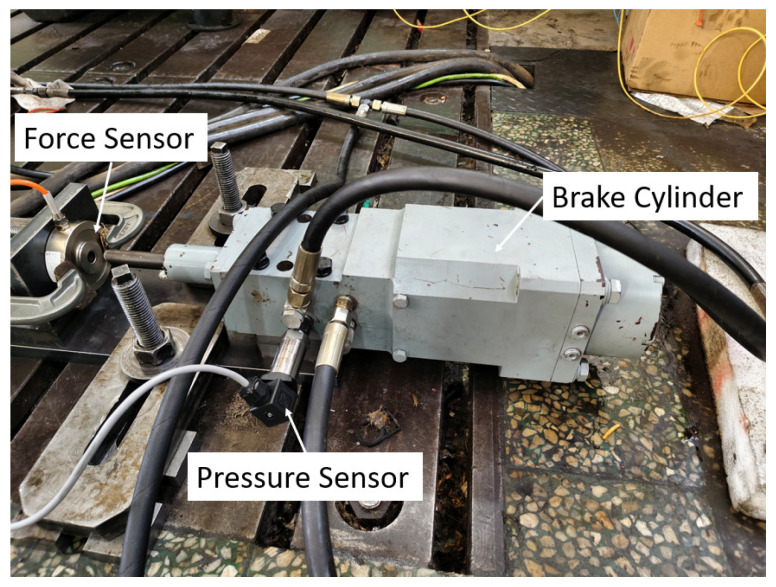
Hydraulic brake cylinder.

**Figure 11:**
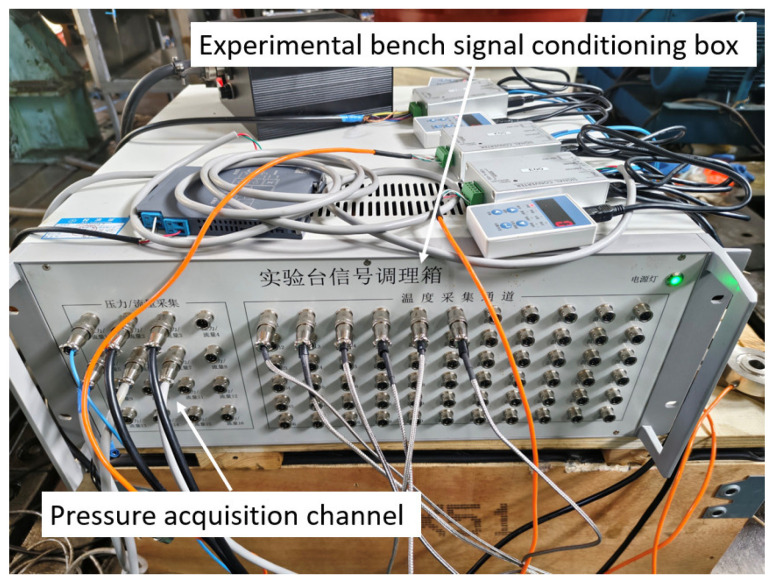
Signal acquisition system.

To verify the accuracy of the simulation results, a test bench was constructed, as shown in Figure 9, including an accumulator, an electric motor, and a hydraulic power unit. Figure 10 shows the brake cylinder, where a pressure sensor is installed at the oil inlet to collect the pressure variation in the brake cylinder in real time. The pressure sensor used is the SUP-P300 from Hangzhou Meiyi Company (Hangzhou, China), with a measurement range of 0–40 MPa, and a maximum measurement error is ±0.5%. The power supply is 24 VDC. Figure 11 shows the signal acquisition system, which acquires data every 50 ms and records and stores it on a computer.

In this study, the braking pressure and its response time were measured, and the simulation accuracy was calculated to verify the accuracy of the simulation results. Each test condition was repeated three times. After the pressure reached a steady state, the average of the three measurements was taken as the final experimental result.

Equation (15) represents the calculation formula for the simulation accuracy of the model.

(15) $$\mathrm{simulation}\;\mathrm{accuracy}=\left(1-\frac{\left|\mathrm{test}\;\mathrm{results}-\mathrm{simulation}\;\mathrm{results}\right|}{\mathrm{test}\;\mathrm{results}}\right)\times 100\%$$

### 5.1. Dynamic Operating Conditions

#### 5.1.1. Proportional Signal

**Figure 12:**
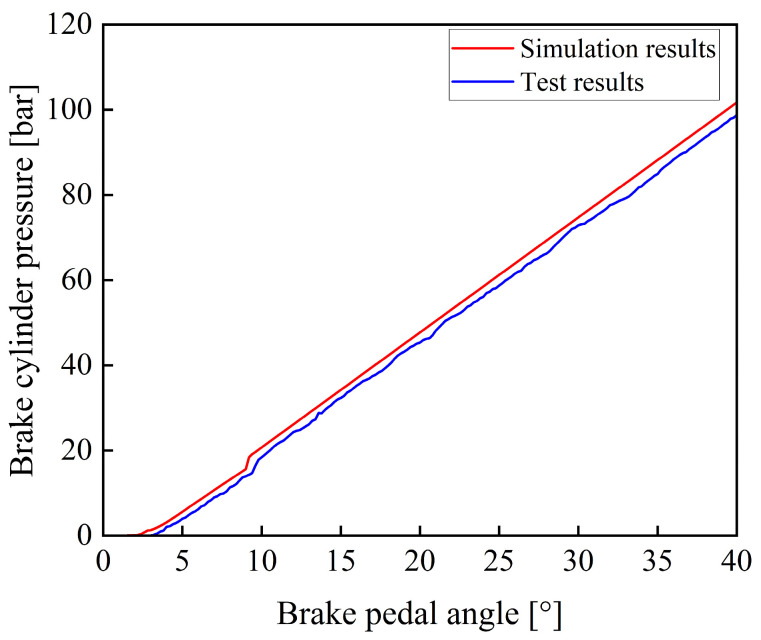
Comparison of simulation results and experimental results under a proportional signal.

During the experiment, the prototype was operated until the operating condition became stable. After the accumulator completed charging and the system pressure stabilized, the braking operation was carried out. By gradually applying the braking command, the pedal rotation angle was increased from 0 to 40° within 2 s, simulating the process of a proportional control signal. The experiment was repeated three times, and the results showed good consistency. Figure 12 shows a comparison between the experimental and the simulation results.

As shown in Figure 12, the experimental and simulation results of the variation in braking pressure with respect to pedal rotation angle exhibit consistent trends. Since certain components were simplified during the model development process, such as by neglecting factors including leakage of the electro-hydraulic proportional valve, the simulated values are slightly higher than the experimental values at the same time.

#### 5.1.2. Trapezoidal Signal

**Figure 13:**
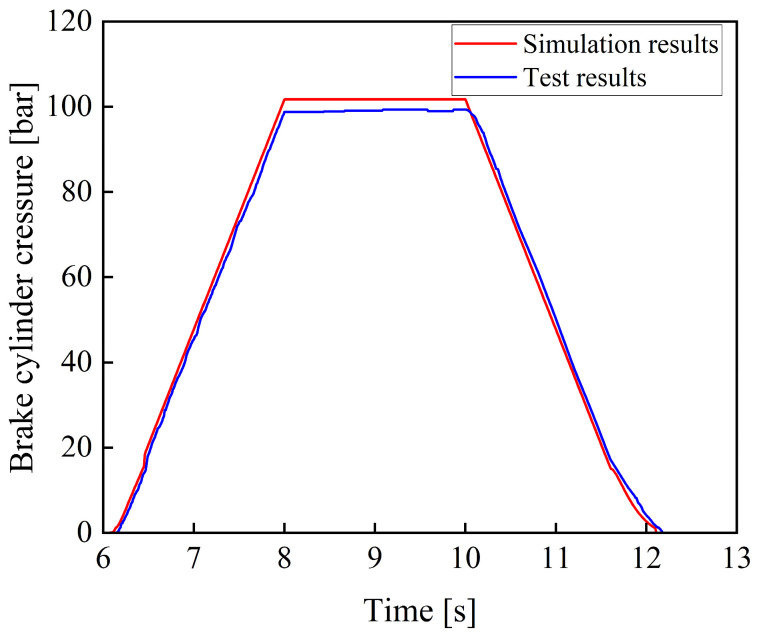
Comparison between simulation and experimental results under a trapezoidal input signal.

Figure 13 shows the comparison between the experimental and the simulation results of the braking pressure under a trapezoidal input signal.

As shown in Figure 13, the experimental results are consistent with the simulation results in terms of variation trends, and no significant deviation is observed, thereby verifying the reliability of the model. Meanwhile, the braking system exhibits good tracking performance with respect to the trapezoidal input signal, and the system response is relatively fast.

### 5.2. Steady-State Operating Conditions

**Figure 14:**
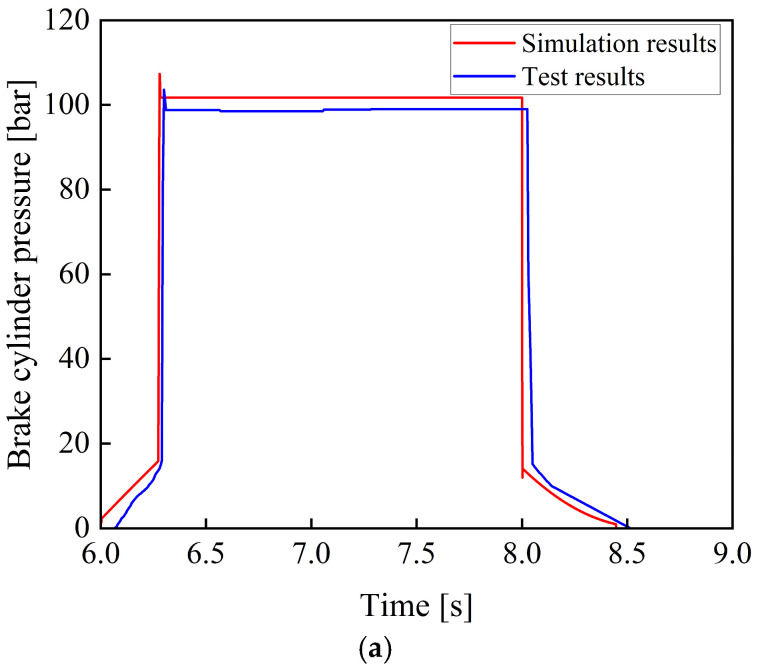

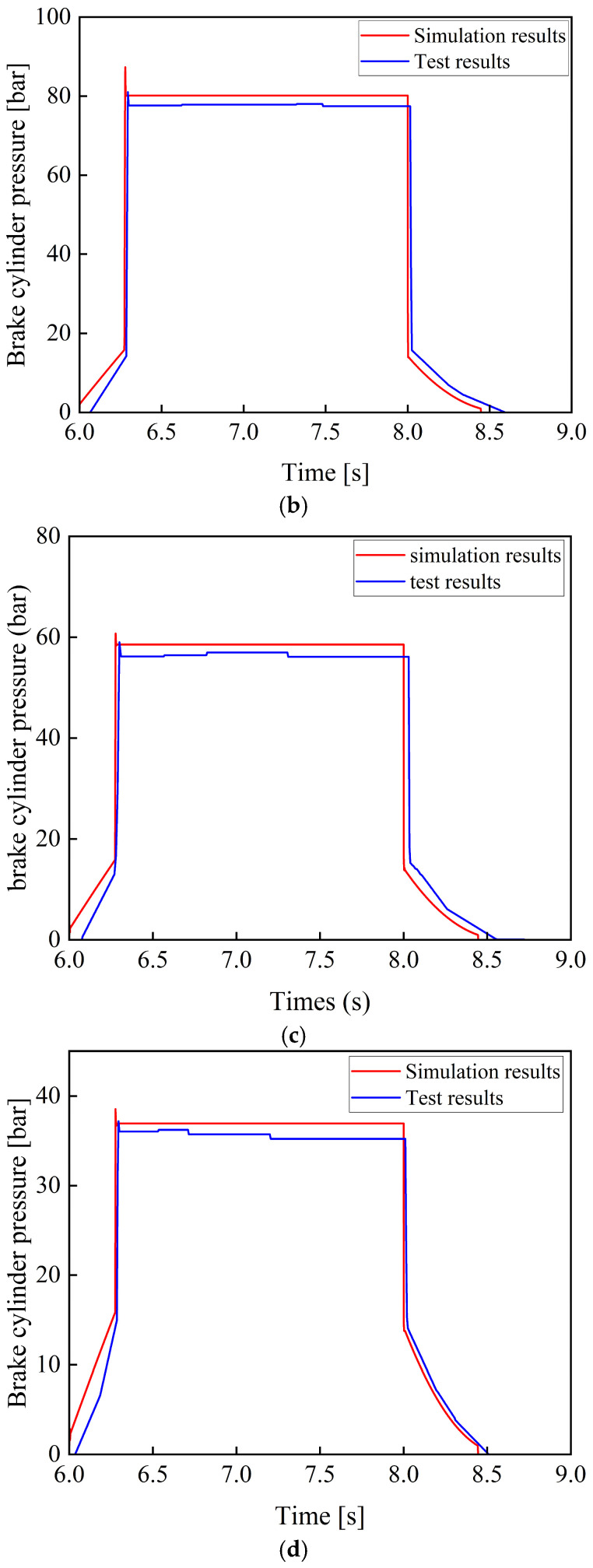
Comparison of simulation and experimental results under different braking percentages. (**a**) Comparison between simulation and experimental results at a braking percentage of 100%. (**b**) Comparison between simulation and experimental results at a braking percentage of 80%. (**c**) Comparison between simulation and experimental results at a braking percentage of 60%. (**d**) Comparison between simulation and experimental results at a braking percentage of 40%.

Figure 14 shows the comparison between the experimental and the simulation results of the braking pressure when the brake pedal is instantaneously fully depressed to simulate a rectangular signal.

As shown in Figure 14, during the processes of system pressure buildup and release, the system response characteristics measured in the experiment are consistent with the simulation results. No significant overshoot occurs during the response process, and the pressure output remains relatively stable without significant oscillations.

As shown in Table 1 and Table 2, under typical braking conditions, when the braking percentage are 100%, 80%, 60%, and 40%, the simulation accuracy of the braking pressure is greater than 96%, and the simulation accuracy of the response time is greater than 95%. Considering factors such as machining and assembly errors in the actual system and the measurement accuracy of the pressure sensor, the established model can be regarded as capable of realistically reflecting the actual braking performance of the tracked vehicle braking system.

### 5.3. Random Operating Conditions

**Figure 15:**
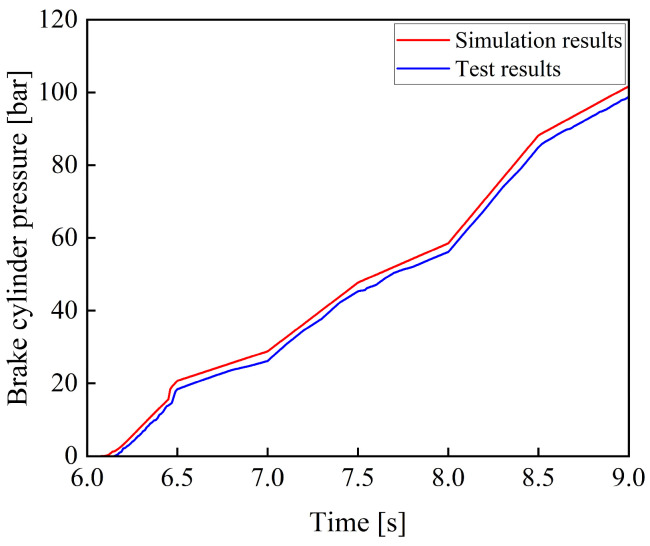
Comparison of simulation and experimental results under random signals.

Figure 15 shows a comparison between the experimental and simulation results of braking pressure under random signals.

As shown in Figure 15, the simulation results are generally consistent with the experimental data in terms of overall trend. The braking system shows a fast response, accurate pressure variation, and good stability, meeting the braking requirements of tracked vehicles under complex operating conditions.

**Figure 16:**
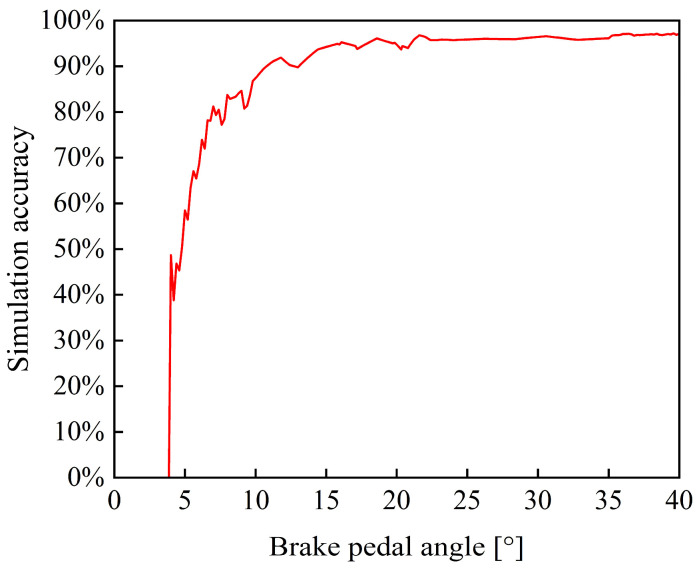
Accuracy of braking pressure simulation under random signals.

Figure 16 shows the simulation accuracy of braking pressure at each pedal angle under random operating conditions. When the input pedal angle is below 7°, the simulation accuracy is less than 80%. However, when the pedal angle exceeds 16° (corresponding to a braking percentage of 40%), the accuracy increases to above 95%. This phenomenon can be attributed to the nonlinear characteristics of the braking system at low input levels. In the initial stage, there is pedal-free travel, during which almost no braking pressure is generated. Furthermore, because the braking pressure is relatively low in this range, a small absolute error results in a large relative error. As the braking percentage increases, the braking pressure is approximately linearly proportional to it, resulting in a more stable pressure response and thus improving the simulation accuracy. Considering that tracked vehicles typically operate under medium- to high-intensity braking conditions, this digital model has high accuracy within its main operating range and is therefore considered to meet the needs of practical applications.

## 6. Influence of Key Component Parameters on Braking Performance

Numerous factors affect the performance of the braking system of tracked vehicles, including the parameters of hydraulic components and operating conditions. These factors have a significant influence on the driving safety of tracked vehicles and the operational reliability of the system. In this study, key components of the braking system, including the accumulator, electro-hydraulic proportional valve, and brake cylinder, are selected as the research objects. The influence of parameter variations in these components on the dynamic response characteristics of the braking system is analyzed, providing a theoretical basis for the optimal design of tracked vehicle braking systems.

### 6.1. Influence of Accumulator Pressure on the Response of the Braking System

**Figure 17:**
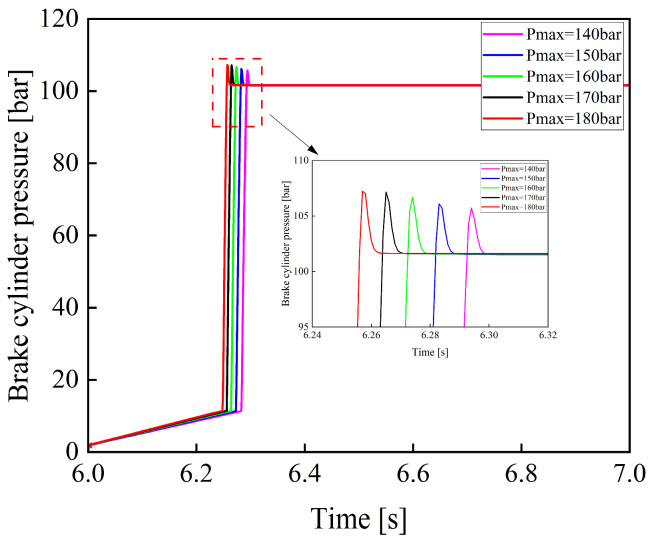
Variation curves of braking pressure under different maximum accumulator charging pressures.

Figure 17 shows the variation curves of brake cylinder pressure when the maximum charging pressure of the accumulator is 140, 150, 160, 170, and 180 bar under a rectangular input signal applied to the electro-hydraulic proportional valve.

As shown in Figure 17, as the maximum charging pressure of the accumulator increases, the response time of the braking system decreases. This is because a higher charging pressure increases the initial pressure difference between the accumulator outlet and the brake cylinder, thereby increasing the initial filling flow rate and accelerating pressure buildup in the brake cylinder. At the same time, it leads to a higher pressure rise rate in the brake cylinder. During the feedback control period of the electro-hydraulic proportional valve, a higher pressure rise rate will inevitably result in greater overshoot, which may cause impacts on the brake cylinder and potentially lead to damage. Conversely, if the maximum charging pressure is too low, the accumulator will undergo frequent charging cycles, thereby increasing energy consumption and reducing its service life. Therefore, an appropriate charging pressure range should be selected in the design of the accumulator to balance system response performance and operational reliability.

### 6.2. Influence of the Diameter of the Electro-Hydraulic Proportional Valve on the Response of the Braking System

**Figure 18:**
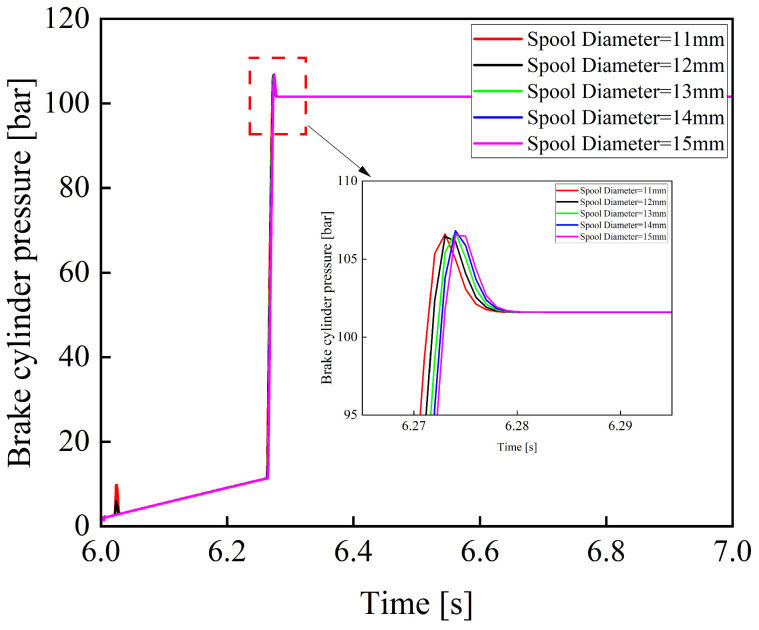
Variation curves of braking pressure under different spool diameters.

Figure 18 shows the variation curves of the brake cylinder pressure when the spool diameter of the electro-hydraulic proportional valve is 11, 12, 13, 14, and 15 mm.

As shown in Figure 18, the diameter of the valve spool in an electro-hydraulic proportional valve has a significant impact on the braking pressure buildup process. A smaller spool diameter leads to noticeable fluctuations during the pressure rise phase. As the spool diameter increases, the effective flow area of the valve orifice increases, and the pressure rise process becomes smoother. Simultaneously, the pressure rise rate also decreases, thereby prolonging the system response time. Therefore, in the structural parameter design of the electro-hydraulic proportional valve, the spool diameter should be appropriately selected to reduce oscillations while minimizing system response time.

### 6.3. Influence of the Brake Cylinder Piston Diameter on the Response of the Braking System

**Figure 19:**
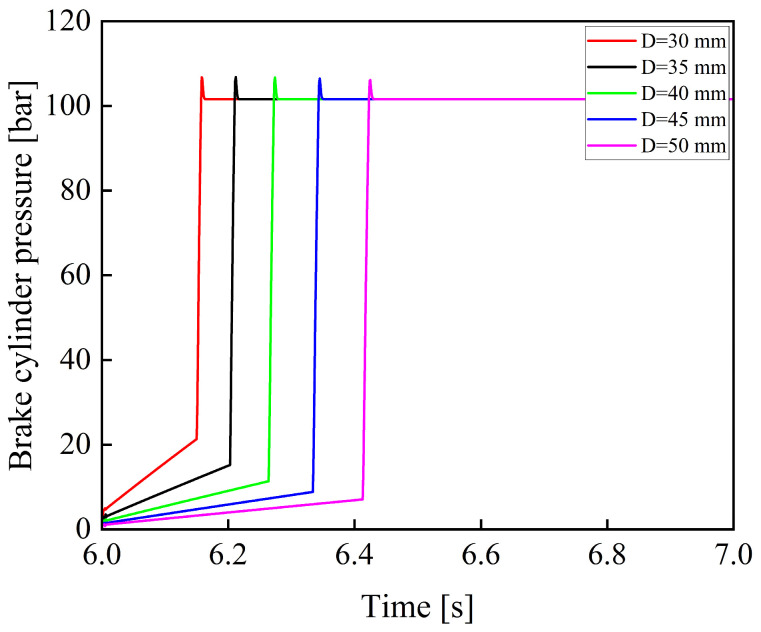
Variation curves of braking pressure under different brake cylinder piston diameters.

Figure 19 shows the variation curves of the brake cylinder pressure when the piston diameter of the brake cylinder is 30, 35, 40, 45, and 50 mm.

As shown in Figure 19, when the piston rod diameter remains constant, the system response time increases significantly as the piston diameter of the brake cylinder increases. This leads to a noticeable increase in braking distance. Therefore, when designing the size of the brake cylinder, the volume of the brake cylinder should be minimized to shorten the system response time and improve overall braking performance, provided that sufficient braking pressure can still be generated.

## 7. Conclusions

Based on the operating principle of the hydraulic braking system of a tracked vehicle, a digital model of the braking system was developed by comprehensively considering factors such as pipeline length. The braking performance under different operating conditions was analyzed, and the following conclusions were drawn:

(1) The braking system is capable of continuously and proportionally generating braking pressure in response to the variation in the pedal rotation angle.
(2) The response time of the braking system is within 0.3 s, enabling effective braking pressure to be established within a short period. During the braking process, no significant overshoot is observed, and the pressure output remains relatively stable without obvious oscillations. During the brake pedal release phase, the hydraulic oil in the brake cylinder can be rapidly discharged into the tank, thereby ensuring normal vehicle operation.
(3) The simulation results of braking pressure are generally consistent with the experimental data in terms of variation trends. Under typical braking conditions, at braking percentages of 100%, 80%, 60%, and 40%, the braking pressure accuracy is above 96%, and the simulation accuracy of response time exceeds 95%. These results indicate that the digital model has high reliability and accuracy and can realistically reflect the braking performance of the system.
(4) The braking pressure closely follows the pedal input signal well, exhibiting accurate and stable pressure variations. When the braking percentage exceeds 40%, the simulation accuracy of braking pressure remains above 95%.
(5) By reasonably designing the parameters of the accumulator and the electro-hydraulic proportional valve, and minimizing the volume of the brake cylinder as much as possible, the braking performance of the system can be improved.

The results indicate that the construction of a high-precision digital model of the braking system enables the prediction and evaluation of braking performance under actual operating conditions. This facilitates parameter analysis and structural optimization at the design stage, thereby improving braking system development efficiency and reducing experimental and development costs.

Although the proposed model and experimental setup have demonstrated satisfactory performance, several aspects can still be further improved. Future work will focus on incorporating additional influencing factors into the model and optimizing key parameters using an orthogonal experimental design to enhance braking performance. Meanwhile, improvements to the experimental setup will also be considered, such as the use of higher-precision sensors and more advanced data acquisition systems to improve measurement accuracy and result reliability.

## Figures and Tables

**Table 1:** Comparison of simulation and experimental results of brake pressure under different braking percentages.

Braking Percentage	Simulation Results/bar	Test Results/bar	Simulation Accuracy
100%	101.73	98.82	97.05%
80%	80.13	77.68	96.85%
60%	58.53	56.42	96.25%
40%	36.94	35.59	96.20%

**Table 2:** Comparison of simulation and experimental results for brake pressure response time under different brake percentages.

Braking Percentage	Simulation Results/s	Test Results/s	Simulation Accuracy
100%	0.279	0.290	96.21%
80%	0.278	0.285	97.54%
60%	0.277	0.290	95.52%
40%	0.277	0.290	95.52%

## Data Availability

The original contributions presented in this study are included in the article. Further inquiries can be directed to the corresponding author.

## Nomenclature

$Q_{pump}$	hydraulic pump output flow [mL/min]
η	hydraulic pump volumetric efficiency [%]
$V_{pump}$	hydraulic pump displacement [mL/r]
n	motor speed [r/min]
$P_{acc0}$	initial absolute pressure of the accumulator [MPa]
$V_{acc0}$	initial inflation volume in the accumulator [L]
$P_{\mathrm{gas}}$	absolute pressure of gas inside the accumulator [MPa]
$V_{gas}$	gas volume inside the accumulator [L]
γ	polytropic exponent of the gas
$P_{accout}$	accumulator oil port pressure [MPa]
$P_{atm}$	atmospheric pressure [MPa]
$Q_{accout}$	accumulator oil port flow rate [L/min]
$F_{m}$	electromagnetic force [N]
p	outlet pressure [MPa]
A	effective piston area of the feedback chamber [mm^2^]
m	spool mass [kg]
x	spool displacement [mm]
$c_{v}$	viscous damping coefficient of the spool [N/(mm/s)]
$c_{s}$	hydrodynamic damping coefficient [N/(mm/s)]
$K_{v}$	stiffness of the return spring [N/mm]
$K_{s}$	hydrodynamic stiffness [N/mm]
$x_{0}$	pre-compression of the return spring [mm]
f	friction force acting on the spool [N]
Q	valve port flow rate [L/min]
$C_{d}$	discharge coefficient of the valve port
A(x)	effective flow area [mm^2^]
$p_{0}$	supply pressure of the hydraulic system [MPa]
ρ	density of the hydraulic oil [kg/m^3^]
$A_{2}$	effective piston area of the brake cylinder [mm^2^]
$m_{2}$	piston mass [kg]
$x_{2}$	piston displacement [mm]
$c_{V2}$	equivalent damping coefficient of the brake cylinder piston [N/(mm/s)]
k	spring stiffness [N/mm]
$x_{20}$	spring pre-compression [mm]
$f_{2}$	friction force acting on the brake cylinder piston [N]
$V_{2}$	Volume of the working chamber of the brake cylinder piston [L]
E	bulk modulus of elasticity of the hydraulic fluid [MPa]
$C_{2}$	leakage coefficient of the brake cylinder [L/MPa/min]
$P_{line}$	outlet pressure of the pipeline [MPa]
$A_{line}$	cross-sectional area of the pipeline [mm]^2^
B	equivalent bulk modulus [MPa]
$E_{wall}$	bulk modulus of elasticity of the pipeline wall material [MPa]
$Q_{line}$	inlet oil flow rate of the pipeline [L/min]
$d_{line}$	pipeline diameter [mm]
L	pipeline length [mm]
θ	vertical inclination angle of the pipeline [°]
$f_{f}$	pipeline resistance coefficient
